# Diet Quality and Change in Blood Lipids during 16 Years of Follow-up and Their Interaction with Genetic Risk for Dyslipidemia

**DOI:** 10.3390/nu8050274

**Published:** 2016-05-09

**Authors:** Emily Sonestedt, Sophie Hellstrand, Isabel Drake, Christina-Alexandra Schulz, Ulrika Ericson, Joanna Hlebowicz, Margaretha M. Persson, Bo Gullberg, Bo Hedblad, Gunnar Engström, Marju Orho-Melander

**Affiliations:** 1 Diabetes and Cardiovascular Disease—Genetic Epidemiology, Department of Clinical Sciences Malmö, Lund University, Jan Waldenströms gata 35, SE-20502 Malmö, Sweden; sophie.hellstrand@med.lu.se (S.H.); isabel.drake@med.lu.se (I.D.); christina-alexandra.schulz@med.lu.se (C.A.S.); ulrika.ericson@med.lu.se (U.E.); marju.orho-melander@med.lu.se (M.O.M.); 2 Experimental Cardiovascular Research Unit, Department of Clinical Sciences Malmö, Lund University, Jan Waldenströms gata 35, SE-20502 Malmö, Sweden; joana.hlebowicz@med.lu.se; 3 Internal Medicine Research Unit, Department of Clinical Sciences Malmö, Lund University, Inga Marie Nilssons gata 32, SE-20502 Malmö, Sweden; margaretha.m.persson@skane.se; 4 Nutritional Epidemiology, Department of Clinical Sciences Malmö, Lund University, Jan Waldenströms gata 35, SE-20502 Malmö, Sweden; bo.gullberg@med.lu.se; 5 Cardiovascular Epidemiology, Department of Clinical Sciences Malmö, Lund University, Jan Waldenströms gata 35, SE-20502 Malmö, Sweden; bo.hedblad@med.lu.se (B.H.); gunnar.engstrom@med.lu.se (G.E.)

**Keywords:** epidemiology, diet, nutrition, genetics, lipids, lipoproteins

## Abstract

A high diet quality according to the Swedish nutrition recommendations is associated with a reduced risk of cardiovascular disease in the population-based Malmö Diet and Cancer cohort. To further clarify this protective association, we examined the association between high diet quality and change in triglycerides, high density lipoprotein-cholesterol (HDL-C), and low density lipoprotein-cholesterol (LDL-C) after 16 years of follow-up in 3152 individuals (61% women; 46–68 years at baseline). In addition, we examined if genetic risk scores composed of 80 lipid-associated genetic variants modify these associations. A diet quality index based on intakes of saturated fat, polyunsaturated fat, sucrose, fiber, fruit and vegetables, and fish was constructed. A high diet quality was associated with lower risk of developing high triglycerides (*p* = 0.02) and high LDL-C (*p* = 0.03) during follow-up compared with a low diet quality. We found an association between diet quality and long-term change in HDL-C only among those with lower genetic risk for low HDL-C as opposed to those with higher genetic risk (*p*-interaction = 0.04). Among those with lower genetic risk for low HDL-C, low diet quality was associated with decreased HDL-C during follow-up (*p* = 0.05). In conclusion, individuals with high adherence to the Swedish nutrition recommendation had lower risk of developing high triglycerides and LDL-C during 16 years of follow-up.

## 1. Introduction

A high diet quality according to the current Swedish nutrition recommendations has previously been associated with decreased risk of cardiovascular disease (CVD) in the Malmö Diet and Cancer (MDC) study cohort [1]. Adherence to the recommendations was assessed using a diet quality index composed of six dietary components: saturated fat, polyunsaturated fat, sucrose, fiber, fruit and vegetables, and fish and shellfish [2]. The diet quality index showed stronger association with CVD risk than the individual index components [1], which points towards the importance of investigating patterns reflecting “whole diet” in relation to disease risk. Dyslipidemia is a major risk factor for CVD, and dietary factors influence blood lipid and lipoprotein concentrations. However, only a few studies have examined the association between food patterns and development of the metabolic syndrome including longitudinal changes in blood lipids and lipoproteins [3,4,5]. To further clarify the protective association of the combined dietary index on CVD risk in the MDC study, we examined its relation to change in blood lipids during 16 years of follow-up. In addition, because genetic variation can influence the response to dietary factors and dietary patterns, we examined if 80 validated lipid- and lipoprotein-associated genetic variants, combined into genetic risk scores, modify these associations. Together, these variants have been estimated to account for 25%–30% of the genetic variance in blood lipid concentrations in Caucasians [6] and can be used as an estimate of the overall genetic susceptibility to dyslipidemia. Several of these genetic loci have previously been associated with changes in total cholesterol or triglycerides during follow-up within the MDC cohort [7].

## 2. Materials and Methods

### 2.1. Study Population and Data Collection

The MDC study is a population-based prospective cohort study with baseline examination between 1991 and 1996 [8,9]. All men born between 1923 and 1945 and all women born between 1923 and 1950 and living in Malmö were invited through public advertisements or personal letters to participate in the study. The cohort comprised of 30,447 individuals participating in at least one part of the baseline examination. The data collection consisted of measuring dietary habits through a modified diet history method (see below) and self-reported information on lifestyle and demographic factors through an extended questionnaire. Nurses measured anthropometrics and blood pressure and collected blood samples (non-fasting). In total, 28,098 individuals completed the dietary collection, the lifestyle questionnaire, and had anthropometrics taken, which gave a participation rate of approximately 40% [8,10]. A random 50% sample of the individuals participating 1992–1994 was invited for an additional visit (after a mean of 0.7 years) where, for example, blood was drawn after an overnight fast for measurements of serum lipids and whole blood glucose (using the hexokinase-glucose-6-phosphate dehydrogenase method). A conversion factor of 1.13 was used to convert whole blood glucose values to plasma glucose values. During the years 2007 to 2012 (after an average of 16 years of follow-up), individuals in this sub-cohort were invited to participate in a re-examination including analyses of fasting blood lipids using the same method as during the baseline, plasma glucose measured using Hemocue (Hemocue, Ängelholm, Sweden), blood pressure measured after 10 min in supine position, a questionnaire on lifestyle factors and anthropometric measurements [11]. The MDC study was approved by the ethical committee at Lund University (LU 51-90), and informed consent was obtained from all participants during recruitment.

For this study, we excluded individuals with diabetes, coronary event, any type of stroke or lipid-lowering medication at baseline. After these exclusions, the study sample comprised of 3152 individuals (61% women, age 46–68 years) with baseline information on dietary intakes, and both baseline and follow-up reexamination with data on blood lipids and lipoproteins, and all of the covariates adjusted for in the analyses.

### 2.2. Dietary Exposure Assessments

The diet assessment instrument used in the MDC study consisted of a combination of a 7-day food diary (*i.e.*, cooked lunch and dinner meals and cold beverages), and a 168-item diet questionnaire (*i.e.*, other foods regularly consumed during the past year with frequencies and usual portion sizes assessed using photographs) [12]. During a 1-h interview, the participants were asked questions about food choices, food preparation practices and portion sizes of the foods consumed during the 7 days.

The diet quality index was developed to reflect the current Swedish nutrition recommendations and dietary guidelines and have been described in detail previously [2]. The index consists of six dietary components: saturated fat (contribution from non-alcohol energy intake; E%), polyunsaturated fat (PUFA, E%), sucrose (E%), fiber (g/MJ), fruit and vegetables (g/day), and fish and shellfish (g/week). For five of the six components, cut-offs were assigned according to current nutrition recommendations: PUFA 5–10 E%, sucrose ≤10 E%, fiber ≥2.4 g/MJ, fruit and vegetables ≥400 g/day and fish and shellfish ≥300 g/week. Because only 2% of the participants had an intake below the recommended level (10 E%) for saturated fat, the cut-off was increased to 14 E% (*i.e.*, approximately one standard deviation [SD] increase). The participants were given one point for each dietary component that reached the recommended intake level, and zero points were given if they were not within the recommended range. The score was divided into three categories: low (0–1 points), medium (2–4 points) and high (5–6 points). The validity and reproducibility of the diet assessment method have been published previously [13,14,15]. The energy-adjusted validation coefficients for the diet method compared to 18 days of weighted food records were as follows: saturated fat (0.56 and 0.68 for men and women, respectively), PUFA (0.26; 0.64), sugar (0.60; 0.74), fiber (0.74; 0.69), fruit (0.60; 0.77), vegetables (0.65; 0.53), and fish (0.35; 0.70) [14,15].

### 2.3. Measurements of Blood Lipids and Lipoprotein Subfractions

Blood lipid levels at baseline and follow-up were analyzed with the same laboratory methods at the Department of Clinical Chemistry at the Skåne University Hospital in Malmö. Blood concentrations of fasting triglycerides and total cholesterol were measured on a DAX 48 automatic analyzer using reagents and calibrators from the supplier of the instrument (Bayer AB, Göteborg, Sweden). High density lipoprotein cholesterol (HDL-C) was determined by the same procedure as used for total cholesterol but after precipitation of low density lipoprotein (LDL) and very low-density lipoprotein (VLDL) with dextran sulphate. LDL-cholesterol (LDL-C) concentration was calculated with the Friedewald formula: LDL-C = total cholesterol − HDL-C − (triglycerides/2.2) among individuals with triglyceride levels below 4.0 mmol/L. Concentrations of lipoprotein subfractions at baseline were measured with the ion mobility method [16,17]. HDL particles were divided into small (76.5–105.0 Å) and large (105.0–145.0 Å) subfractions. LDL particles were divided into very small (LDL 3b, 4a, 4b and 4c; 180.0–204.9 Å), small (LDL 3a, 204.9–214.1 Å), medium (LDL 2b; 214.1–224.6 Å), and large (LDL 2a and 1; 224.6–233.3 Å) subfractions. Intermediate-density lipoprotein (IDL) particles were divided into small (233.3–250.0 Å) and large (250.0–296.0 Å) subfractions. VLDL particles were divided into small (296.0–335.0 Å), medium (335.0–424.0 Å), and large (424.0–547.0 Å) subfractions. Particle diameter of the major LDL peak was also determined. At follow-up, 881 individuals (28%) reported using lipid-lowering medication; 870 individuals used statins or other LDL-lowering medication (Crestor, Lipitor, Pravachol, Zocord or Ezetrol) and 15 individuals used fibrates (Lopid). Correction for lipid-lowering medication at follow-up was performed by adding a constant to the respective lipid levels (statins: +0.208 mmol/L for triglycerides, −0.060 for HDL-C and +1.290 for LDL-C; fibrates: +0.645 for triglycerides, −0.153 for HDL-C and +1.037 for LDL-C) [18]. The Adult AHA/NHLBI statement for the metabolic syndrome was used to identify individuals with high plasma triglyceride concentrations (≥1.7 mmol/L and/or triglyceride lowering treatment) and low HDL-C (using corrected values; <1.0 mmol/L for men and <1.3 mmol/L for women) [19]. High LDL-C was defined as above 4.1 mmol/L and/or lipid lowering treatment [20].

The AHA/NHLBI criteria were also used to identify individuals with elevated waist circumference (≥102 cm for men and ≥88 for women), hypertension (≥130 mmHg SBP or ≥85 mmHg DBP or antihypertensive drug treatment), and elevated plasma glucose (≥5.6 mmol/L or glucose lowering drug treatment) [19].

### 2.4. Genetic Factors

All SNPs (*n* = 91) that reached the genome-wide significance level for triglycerides, HDL-C or LDL-C in the study by Teslovich *et al.* [6] were genotyped except the *LPA* rs1084651, *JMJD1C* rs10761731 and *NPC1L1* rs217386 because of difficulties in genotyping or a lack of proxies available. Genotyping was performed at the Clinical Research Centre, Malmö, Sweden, using Sequenom MassARRAY (Sequenom, San Diego, CA, USA) or Taqman allelic discrimination on an ABI 7900 (Applied Biosystems, Foster City, CA, USA). For the current study, SNPs were then excluded if the genotyping success rate was less than 90% (*i.e.*, *COBLL1* rs10195252, *KLF14* rs4731702, *PLEC1* rs11136341 and *ABCA8* rs4148008) or if the Hardy-Weinberg equilibrium p-value was less than 0.00057 (0.05/87) (*i.e.*, *ANGPTL3* rs2131925, *TYW1B* rs13238203, *SCARB1* rs838880, *OSBPL7* rs7206971, *LILRA3* rs386000, *PLTP* rs6065906 and *MOSC1* rs2642442). Weighted genetic risk scores were constructed using PLINK (version 1.07) for triglycerides (26 SNPs), HDL-C (41 SNPs) and LDL-C (32 SNPs) by multiplying the effect size (*i.e.*, β-coefficients) observed in the meta-analysis [6] by the number of risk alleles and then summing the products (Table S1). Genetic risk scores were calculated for those 3094 individuals with more than 60% of the SNPs successfully genotyped.

### 2.5. Other Variables

Subjects, wearing light clothes and no shoes, were weighed using a balance-beam scale for weight and a fixed stadiometer for height. Alcohol habits were categorized into six groups. Individuals reporting no alcohol consumption during the last year in the questionnaire, and also no consumption in the 7-day food diary, were categorized as zero-consumers of alcohol. The other individuals were divided into gender-specific quintiles based on their alcohol consumption in the food diary. Smoking habits were categorized into three groups: smokers (including irregular smokers), ex-smokers and never smokers. Education was categorized into five groups based on the highest level of education attained: elementary or less, primary and secondary, upper secondary, further education without a degree, and university degree. A leisure-time physical activity score was constructed taking into account the intensity and duration spent on 17 different activities. The score was divided into quintiles. Non-adequate reporters of energy were identified by comparing their reported energy intake with their total energy expenditure (*i.e.*, estimated from their calculated basal metabolic rate and self-reports of leisure-time physical activity, work activity, household work, and sleep hours). Individuals with reported energy intake above or below the 95% confidence interval (CI) for total energy expenditure were categorized as “misreporters”. Individuals answering yes to the questionnaire item “Have you substantially changed your dietary habits in the past?” were classified as “dietary changers”.

### 2.6. Statistical Analyses

The Statistical Package of the Social Science (SPSS, version 20; IBM Corporation, Armonk, NY, USA) was used for statistical analyses. Differences in participant characteristics between attendees and the two groups of non-attendees were tested through t-tests for continuous variables and χ2 test for categorical variables. General Linear Model was used to estimate the association between diet quality index and clinical risk factors at baseline adjusted for age, sex (if applicable), season, total energy intake, education, smoking, leisure-time physical activity, alcohol consumption and waist circumference. We also examined the associations with blood lipid concentrations at follow-up (with additional adjustments for follow-up time) and change in concentrations (delta-values) during the follow-up (with additional adjustments for follow-up time, and ln-transformed baseline lipid concentrations). Changes in waist circumference and smoking status during follow-up were included as covariates in an additional model. Trends across diet index categories were tested with diet quality index as continuous variable (0 to 6) and ln-transformed clinical risk factors. The combined diet quality index was the main exposure, but we also examined the associations with adherence to each component separately. Logistic regression was used to examine the association between diet quality index categories and risk of developing high triglycerides (≥1.7 mmol/L), low HDL-C (<1.0 mmol/L for men; <1.3 mmol/L for women) or high LDL-C (>4.1 mmol/L or lipid lowering medication) at re-examination if having normal blood lipid levels at baseline. As secondary outcomes, we also examined the association between diet quality index categories and risk of developing elevated waist circumference (≥102 cm for men and ≥88 for women), hypertension (≥130 mmHg SBP or ≥85 mmHg DBP or antihypertensive drug treatment), or elevated plasma glucose (≥5.6 mmol/L or glucose lowering drug treatment) at re-examination if having normal levels at baseline. These analyses were adjusted for age, sex (if applicable), season, total energy intake, education, smoking, leisure-time physical activity, alcohol consumption, and waist circumference. Changes in waist circumference and smoking status during follow-up were included as covariates in an additional model. All analyses were also carried out separately in men and women due to differences in dietary habits and blood lipid- and lipoprotein concentrations between men and women. In sensitivity analysis, we excluded potential “misreporters” of energy and ”dietary changers” (for definition see under “other variables”).

Interaction analyses between diet quality index and the three genetic risk scores on change in lipid- and lipoprotein concentrations were tested by introducing an interaction term using continuous variables and adjusted for sex, age, follow-up time, baseline blood lipid concentrations, season, total energy intake, education, smoking, leisure-time physical activity, alcohol consumption and waist circumference.

## 3. Results

### 3.1. Descriptive Analyses

Individuals not attending the re-examination generally had higher waist circumference, lower diet index score, worse lipid profile and were more often smokers at baseline compared to attendees (Table S2). A high diet quality was positively associated with age, but not with BMI. In addition, among individuals with high index scores the frequency of smokers and individuals with low education was lower and the frequency of individuals with high physical activity was higher compared to individuals with low index scores (Table 1). At follow-up, 28% (*n* = 881) of all individuals were using lipid-lowering medication and this frequency was similar in all diet index groups. During the 16 years of follow-up, the average triglyceride concentration in the whole study sample had decreased from 1.25 (SD ± 0.60) mmol/L to 1.07 ± 0.50 mmol/L (values corrected for lipid-lowering medication: 1.13 ± 0.52 mmol/L), and LDL-C from 4.12 ± 0.97 mmol/L to 3.32 ± 0.93 mmol/L (corrected values: 3.68 ± 0.80 mmol/L). The average HDL-C concentration was 1.42 ± 0.37 mmol/L at baseline compared with 1.44 ± 0.43 mmol/L (corrected values 1.42 ± 0.44 mmol/L) at follow-up. During the follow-up, the average BMI had increased from 25.3 ± 3.6 kg/m^2^ to 26.7 ± 4.3 kg/m^2^ and waist circumference from 78.1 ± 10.7 cm to 87.9 ± 12.0 cm in women and 93.9 ± 10.3 cm to 99.0 ± 10.2 cm in men. The frequency of smokers was 23% at baseline compared with 9% at follow-up.

### 3.2. Cross-Sectional Analyses of Association between Diet Quality Index and Blood Lipids and Lipoproteins

The diet quality index was only marginally associated with blood lipid- and lipoprotein concentrations at baseline (Table 1). However, among individuals with a high diet quality, we generally observed a better lipoprotein profile (*i.e.*, higher HDL-C (*p* = 0.02) and lower large LDL (*p* = 0.046), medium VLDL (*p* = 0.04) and large VLDL (*p* = 0.01) in analyses with men and women combined; Table S3) compared to those with low diet quality. In addition, among women with high diet quality, we observed 35% lower odds of having low HDL-C concentrations at baseline compared with those with low diet quality (Table S4). This association was even stronger after excluding individuals with unstable food habits (diet changers) and misreporters (OR, 0.59; 95% CI: 0.34–1.03; *p*-trend: 0.004). After these exclusions, we also observed a similar trend of association among men (OR, 0.58; 95% CI: 0.29–1.16; *p*-trend: 0.08).

The associations between diet quality index and blood lipid concentrations were rather strong even after 16 years of follow-up (Table 2). After excluding diet changers and misreporters, all associations between the diet quality index and standard blood lipids were statistically significant (*p*-values for triglycerides: 0.008; HDL-C: 0.006; and LDL-C: 0.03).

### 3.3. Longitudinal Analysis of Association between Diet Quality at Baseline and Blood Lipids

We found only minor associations between diet quality index and change in blood lipids during follow-up (delta-values) (Table 2). After excluding diet changers and misreporters, those with a high diet quality had a more pronounced decrease in triglyceride (*p* = 0.03) and LDL-C (*p* = 0.02) concentrations compared to those with low diet quality (Table 2). Additional adjustments for change in waist circumference and smoking habits during follow-up only slightly attenuated these associations (*p* = 0.10 and 0.03 for triglycerides and LDL-C, respectively).

Diet quality index was inversely associated with risk of developing high triglycerides and LDL-C. Among those with normal triglyceride levels at baseline (82% of the population), 11% had developed hypertriglyceridemia in the low diet quality group and 6% in the high diet quality group. The risk of developing hypertriglyceridemia during follow-up was 46% lower (95% CI: 5%–69%) among those with a high compared with a low diet quality (Table 3). The risk estimates were stronger after excluding diet changers and misreporters. However, adjusting for waist circumference and smoking habits did not affect the results. Diet quality was not associated with risk of developing elevated waist circumference. Among those without hypertension at baseline (23.7% of the population), 56% had developed hypertension in the low diet quality group and 66% in the high diet quality group. In addition, we found an increased risk of developing elevated plasma glucose during follow-up with high diet quality. The association did not, however, remain after excluding diet changers and misreporters (Table 3).

### 3.4. Association between Dietary Components Included in the Diet Quality Index and Blood Lipids

The individuals with a low intake of sucrose had higher HDL-C concentrations both at baseline (*p* = 3 × 10^−7^) and at follow-up (*p* = 0.0001) compared with those that had an intake higher than the recommendation for sucrose (Table S5). In addition, those with low sucrose intake had lower triglycerides levels at baseline but not at follow-up (*p* = 0.18). Those that reached the recommended intake for fiber showed stronger decrease in triglycerides concentrations during follow-up (on average −0.15 mmol/L) compared with those with a lower fiber intake (on average −0.11 mmol/L) (Table S5). After excluding misreporters and dietary changers, these findings remained statistically significant and were generally stronger (Table S5). Similar results were observed for men and women, especially for the associations between dietary components and triglycerides and HDL-C (Table S6).

### 3.5. Interaction with Genetic Risk for Dyslipidemia

The respective genetic risk scores explained 7.3% of the variance in LDL-C, 5.3% of the variance in HDL-C and 4.0% of the variance in triglycerides. The genetic risk scores were also significantly associated with longitudinal changes in the corresponding trait (*p* = 1 × 10^−8^, 1 × 10^−4^, and 0.003, for TG, HDL-C and LDL-C, respectively). We found a nominal significant interaction between diet quality index and genetic risk score for low HDL-C on change in HDL-C during the follow-up (*p* = 0.04) (Table 4). Specifically, we found association between diet quality and HDL-C change during follow-up among those with lower genetic risk for low HDL-C but not among those with higher genetic risk; among those with low genetic risk for low HDL-C, low diet quality was associated with decreased HDL-C during follow-up.

## 4. Discussion

In this population-based prospective cohort, we found that individuals with high adherence to the Swedish nutrition recommendation had higher HDL-C concentrations at baseline and lower risk of developing high triglycerides and LDL-C during the follow-up. A few studies have examined the association between dietary pattern and development of dyslipidemia. For example, an increase in diet score according to the French dietary guidelines was associated with lower 6-year risk of metabolic syndrome [3]. Among middle-aged and older individuals in the Framingham Heart Study Offspring Cohort, higher Mediterranean-style dietary pattern score was associated with lower triglycerides and higher HDL-C after a mean of 7 years of follow-up after adjustment for baseline values [4]. Men with high adherence to the healthy eating index had smaller change in triglyceride concentrations during 6.7 years than those with low adherence in the Tehran Lipid and Glucose Study of 469 adults [5]. A connection between high consumption of soft drinks and increased risk of incident dyslipidemia has been observed in several long-term observational studies [21,22]. In cross-sectional analyses, adherence to dietary guidelines for Americans was inversely related to triglyceride concentration, but not HDL-C, in the Framingham Heart Study [23]. Similar association was found in a French study with adherence to the French dietary guidelines [24]. Although the focus in this study was on change in blood lipids and lipoproteins, in post-hoc analyses we analyzed the risk of developing elevated waist circumference, hypertension and elevated plasma glucose. However, changes in these intermediate factors could not explain the decreased CVD risk among individuals with high quality diet. The increased risk of elevated blood glucose with high diet quality was attenuated after excluding diet changers and misreporters, indicating that those that developed elevated blood glucose might have changed their diet because of illness before the baseline examination. These results also point towards the importance of using disease-specific dietary indices. For example, the optimal fat intake might differ when studying risk of CVD compared with type 2 diabetes [25]. It might also be problematic to investigate outcomes with very high occurrence due to low statistical power. For example, we observed a high frequency of hypertension at baseline (76.3% of the study population) with 65% of those without hypertension at baseline developing hypertension during follow-up.

As often seen in observational cohort studies, the included study sample comprised a healthier population than the general population. The individuals included in the current study had to visit the study center at three occasions during the baseline examinations. In addition, they had to be alive 16 year later and visit the study center again at the re-examination. The individuals that participated in the baseline examination but not at re-examination were more often smokers, had higher BMI and blood pressure and had a higher prevalence of diabetes [11]. They also had a lower diet index score and worse blood lipid profile. Such survival bias may contribute to the fewer individuals than expected in our longitudinal study sample with severe dyslipidemia or high genetic risk for dyslipidemia. This may have introduced a narrower range in blood lipid and lipoprotein concentrations, and probably also a narrower range in dietary intakes, and therefore may have reduced the likelihood of observing differences between diet quality and risk of dyslipidemia or interactions between genetic risk and diet quality. In addition, the individuals in the cohort were middle-aged and older (46–68 years at baseline), which may limit the possibility to observe associations and interactions. Another study within the MDC study showed that favorable changes in LDL-C and HDL-C during follow-up decreased the atherosclerotic process, measured by intima media thickness (IMT) progression rate in the common carotid artery [11]. In addition, although similar methods for blood lipid measurements were used at the two occasions, it is always important to reflect on the precision in baseline *vs.* re-examination in lipid measurements. Further, use of lipid-lowering medication increased markedly during the follow-up being at a much higher level at the follow-up visit (*i.e.*, 2007–2012) as compared to at baseline (*i.e.*, 1992–1994). We have, however, used an established formula to correct the blood lipid concentrations among individuals using lipid-lowering medication [18].

Dietary habits were only reported at the baseline examination, and we were not able to account for any changes in dietary intakes that may have occurred during the follow-up. However, we observed rather stable food habits using the MDC method one year apart with correlation coefficients generally above 0.70 [13]. In our study, those with recommended low intakes of sucrose had lower triglycerides and higher HDL-C concentrations at baseline compared to those with high sucrose intakes. After 16 years, we could still see a significant difference in HDL-C between those that followed the recommendation for sucrose at baseline and those that did not follow the recommendation. This observation may indicate that the food habits are rather stable in this population. We additionally observed associations between the diet quality index and blood lipids at follow-up only after excluding misreporters and those reporting dietary change. Such individuals may have further changed their diet during follow-up, introducing misclassification of the dietary exposure, and therefore may contribute to limit the possibility to see any associations if included. This observation highlights the importance of excluding misreporters in the analyses and asking the participants about the stability of their food habits, especially if the food habits are only collected once.

Fiber was the only included diet component that significantly associated with change in blood lipids. The relative validity of the diet method used in the MDC study is generally high. Importantly, the dietary instrument used in the MDC study was specifically developed to estimate fiber intake in this middle-aged population. Although we have adjusted for several confounders, including change in waist circumference and smoking habits during the follow-up, it might be that the diet quality index correlates with other confounding factors that are of importance for the change in blood lipid concentrations.

In this study, we examined if genetic susceptibility to dyslipidemia (by combining 80 validated genetic variants associated with blood lipids) modifies the association between the diet quality index and changes in blood lipid concentrations. By stratifying the genetic risk for low HDL-C, we observed a decrease in HDL-C during follow-up with low diet quality only among those with low genetic risk for low HDL-C. However, overall we found no strong evidence that genetic risk for dyslipidemia would significantly modify the association between the diet quality index and change in blood lipids, especially if we take multiple testing into account. Previously, in the randomized controlled trial of the Diabetes Prevention Program (DPP), an intensive weight-loss lifestyle intervention with a focus on reducing fat intake among overweight individuals with pre-diabetes, an attenuated LDL lowering effect (especially small LDL) was observed among those with a high genetic risk score composed of 32 lipid-associated SNPs [26]. The genetic variants included in genetic risk scores are associated with different mechanisms of lipid- and lipoprotein metabolism and it may therefore be important to examine genetic variants affecting specific mechanisms and pathways separately to address whether genetic susceptibility affecting such specific mechanisms or pathways would modify the associations. We previously observed that within the MDC cohort, *APOE* rs4420638 was associated with a change in total cholesterol, and *TRIB1* rs2954029 and *APOA1* rs6589564 were associated with a change in triglyceride concentrations during the follow-up time [7]. In addition, it would be important to identify novel loci that interact with dietary habits. For example, genetic markers have been identified that associate with triglyceride response after a high-fat diet [27].

## 5. Conclusions

Diet quality reflected by adherence to the Swedish nutrition recommendations and dietary guidelines has previously shown a protective association with CVD risk. By also examining the risk of developing dyslipidemia and other intermediate risk factors, we can obtain insights into the potential mechanisms behind the increased risk of CVD. The results from the present study indicate that this association may partly be explained through changes in blood lipids. We found no strong evidence that genetic risk for dyslipidemia would modify the association between diet quality index and change in blood lipids over time.

## Figures and Tables

**Table 1:** Participant characteristics, dietary intakes and clinical risk factors at baseline in categories of diet quality index in 1222 men and 1930 women.

Variables	Men				Women			
	Low (0–1)	Medium (2–4)	High (5–6)	*p*-Trend *	Low (0–1)	Medium (2–4)	High (5–6)	*p*-Trend *
Individuals, *n*	150	898	174		229	1323	378	
Age, year	55.2 (5.5)	56.6 (5.9)	56.6 (5.7)	0.05	55.7 (5.7)	56.3 (5.6)	56.7 (5.7)	0.03
Smokers at baseline, %	33.3%	23.8%	13.8%		32.3%	23.4%	14.3%	
Smokers at FU, %	16.9%	9.7%	4.8%		16.3%	8.7%	5.7%	
High physical activity, %	18.7%	20.3%	27.6%		13.5%	19.7%	24.9%	
Low education, %	44.7%	45.8%	35.1%		45.9%	39.3%	34.1%	
University degree, %	16.7%	11.4%	12.1%		8.3%	13.2%	18.0%	
Lipid-lowering medication at FU, %	30.7%	31.0%	32.2%		27.9%	24.5%	27.0%	
Energy intake, MJ	11.8 (2.8)	11.6 (2.9)	10.8 (2.7)	0.001	8.9 (2.1)	8.7 (2.1)	8.5 (1.8)	0.01
Alcohol intake, g/day	12.3 (12.5)	16.1 (14.8)	14.6 (12.2)	0.20	6.7 (7.8)	7.7 (7.6)	7.3 (7.1)	0.53
Saturated fat, E%	18.4 (3.8)	16.6 (3.7)	12.5 (2.3)	<0.001	18.0 (3.4)	16.1 (3.6)	13.0 (2.3)	<0.001
Polyunsaturated fat, E%	5.4 (1.8)	6.4 (1.6)	6.2 (1.1)	<0.001	5.0 (1.4)	5.9 (1.5)	6.2 (1.2)	<0.001
Sucrose, E%	11.2 (4.1)	7.6 (3.0)	7.1 (2.2)	<0.001	11.6 (4.0)	8.5 (2.9)	7.7 (1.9)	<0.001
Fiber, g/MJ	1.7 (0.4)	2.1 (0.5)	2.9 (0.6)	<0.001	1.9 (0.4)	2.4 (0.6)	3.0 (0.6)	<0.001
Fruit and vegetables, g/day	248 (93)	344 (157)	560 (165)	<0.001	279 (105)	405 (169)	571 (156)	<0.001
Fish and shellfish, g/week	166 (147)	338 (247)	480 (373)	<0.001	176 (142)	295 (200)	453 (221)	<0.001
BMI, kg/m^2^	25.7 (0.3)	25.5 (0.1)	25.5 (0.3)	0.84 (0.23)	24.5 (0.3)	24.7 (0.1)	24.8 (0.2)	0.16 (0.25)
Waist circumference, cm	91.9 (0.8)	91.3 (0.4)	90.4 (0.7)	0.51 (0.81)	75.4 (0.7)	75.9 (0.3)	75.7 (0.5)	0.62 (0.64)
Diastolic blood pressure, mmHg	87.9 (0.8)	87.4 (0.4)	86.6 (0.7)	0.61 (0.36)	84.4 (0.6)	84.5 (0.3)	84.5 (0.5)	0.64 (0.92)
Systolic blood pressure, mmHg	140.3 (1.5)	138.9 (0.8)	138.5 (1.4)	0.97 (0.75)	135.9 (1.2)	136.0 (0.6)	136.7 (1.0)	0.33 (0.49)
Plasma glucose, mmol/L	5.84 (0.08)	5.83 (0.04)	5.67 (0.08)	0.06 (**0.02**)	5.49 (0.05)	5.45 (0.02)	5.45 (0.04)	0.82 (0.16)
Plasma insulin, pmol/L	59.1 (3.8)	55.6 (2.0)	53.0 (3.7)	0.054 (0.13)	44.4 (2.1)	42.2 (1.1)	44.3 (1.7)	0.58 (0.38)
Total cholesterol, mmol/L	6.04 (0.08)	5.96 (0.05)	5.96 (0.08)	0.50 (0.32)	6.17 (0.08)	6.13 (0.04)	6.24 (0.06)	0.12 (0.60)
Triglycerides, mmol/L	1.48 (0.05)	1.35 (0.03)	1.41 (0.05)	0.50 (0.54)	1.20 (0.04)	1.20 (0.02)	1.17 (0.03)	0.35 (**0.03**)
HDL-C, mmol/L	1.18 (0.02)	1.22 (0.01)	1.20 (0.02)	0.42 (0.20)	1.50 (0.02)	1.51 (0.01)	1.55 (0.02)	**0.03 (0.006)**
LDL-C, mmol/L	4.18 (0.08)	4.12 (0.04)	4.11 (0.07)	0.36 (0.18)	4.12 (0.07)	4.07 (0.04)	4.16 (0.06)	0.31 (0.96)
HDL-small, nmol/L	2366 (163)	2515 (94)	2847 (155)	0.06 (0.12)	2916 (129)	2926 (68)	2928 (104)	0.49 (0.66)
HDL-large, nmol/L	1088 (79)	1214 (45)	1261 (75)	0.28 (0.15)	1960 (82)	1951 (43)	1990 (66)	0.32 (0.34)
LDL-very small, nmol/L	123.2 (5.5)	112.5 (3.2)	106.1 (5.2)	0.24 (0.77)	96.5 (3.6)	96.2 (1.9)	99.1 (2.9)	0.80 (0.40)
LDL-small, nmol/L	107.4 (7.2)	90.3 (4.1)	84.9 (6.9)	0.15 (0.31)	63.7 (3.8)	62.4 (2.0)	64.2 (3.0)	0.43 (0.87)
LDL-medium, nmol/L	149.9 (8.1)	141.4 (4.7)	134.4 (7.7)	0.29 (0.23)	105.3 (5.0)	98.6 (2.6)	99.1 (4.0)	0.20 (0.94)
LDL-large, nmol/L	446 (16)	458 (9)	430 (16)	0.20 (0.055)	432 (12)	407 (6)	395 (10)	0.09 (0.61)
IDL-small, nmol/L	121 (4)	119 (2)	114 (8)	**0.046 (0.03)**	117 (3)	113 (2)	113 (3)	0.92 (0.41)
IDL-large, nmol/L	183 (9)	190 (5)	179 (8)	0.35 (0.31)	239 (9)	237 (5)	235 (7)	0.57 (0.18)
VLDL-small, nmol/L	50.1 (1.7)	50.8 (1.0)	49.2 (1.6)	0.12 (0.09)	55.5 (1.6)	52.8 (0.8)	53.1 (1.3)	1.00 (0.60)
VLDL-medium, nmol/L	36.5 (1.3)	35.9 (0.8)	34.5 (1.3)	0.053 (0.07)	36.7 (1.1)	34.2 (0.6)	33.6 (0.9)	0.18 (0.32)
VLDL-large, nmol/L	9.82 (0.45)	9.54 (0.26)	9.51 (0.43)	0.27 (0.25)	9.48 (0.37)	8.62 (0.19)	8.24 (0.30)	**0.01 (0.01)**
LDL peak diameter, nm	217.1 (0.5)	218.2 (0.3)	218.0 (0.5)	0.98 (0.92)	222.0 (0.4)	222.0 (0.2)	222.3 (0.3)	0.47 (0.40)

General linear model was used to calculate adjusted mean and SE; The analyses were adjusted for age, season, total energy intake, smoking, education, leisure-time physical activity, alcohol habits, and waist circumference; Abbreviations: IDL, intermediate-density lipoproteins; VLDL, very low density lipoprotein; FU, follow-up; * *p*-Trend was calculated with diet index as continuous variable and ln-adjusted dependent variables. *p*-Values in parentheses: results excluding individuals reporting dietary changes in the past and misreporters of energy (*n* = 2001 remained).

**Table 2:** Blood lipids at follow-up and change in blood lipids during follow-up (mean and 95% CI) by categories of diet index.

Traits	Subjects	Low (0–1)	Medium (2–4)	High (5–6)	*p*-Trend *
Triglycerides at follow-up ^†^	All	1.19 (1.13, 1.24)	1.12 (1.09, 1.15)	1.15 (1.10, 1.19)	0.07 (0.008)
	Men	1.22 (1.13, 1.31)	1.11 (1.06, 1.16)	1.18 (1.10, 1.27)	0.22 (0.04)
	Women	1.15 (1.09, 1.22)	1.12 (1.08, 1.15)	1.12 (1.06, 1.17)	0.17 (0.08)
HDL-C at follow-up ^†^	All	1.38 (1.34, 1.42)	1.42 (1.40, 1.44)	1.41 (1.38, 1.45)	0.10 (0.006)
	Men	1.16 (1.10, 1.22)	1.24 (1.20, 1.27)	1.19 (1.13, 1,25)	0.52 (0.07)
	Women	1.52 (1.46, 1.58)	1.54 (1.51, 1.57)	1.55 (1.51, 1.60)	0.11 (0.04)
LDL-C at follow-up ^†^	All	3.65 (3.57, 3.74)	3.67 (3.62, 3.71)	3.61 (3.54, 3.68)	0.30 (0.03)
	Men	3.49 (3.36, 3.62)	3.54 (3.47, 3.61)	3.43 (3.31, 3.55)	0.20 (0.08)
	Women	3.75 (3.64, 3.87)	3.74 (3.68, 3.80)	3.71 (3.62, 3.81)	0.80 (0.18)
∆Triglycerides ^‡^	All	−0.09 (−0.14, −0.04)	−0.13 (−0.16, −0.11)	−0.11 (−0.15, −0.07)	0.11 (0.03)
	Men	−0.20 (−0.28, −0.12)	−0.26 (−0.31, −0.22)	−0.22 (−0.30, −0.14)	0.42 (0.07)
	Women	−0.02 (−0.08, 0.04)	−0.06 (−0.09, −0.03)	−0.05 (−0.09, 0.002)	0.14 (0.28)
∆HDL-C ^‡^	All	−0.01 (−0.04, 0.02)	0.01 (−0.004, 0.03)	−0.003 (−0.03, 0.02)	0.43 (0.18)
	Men	−0.04 (−0.08, 0.01)	0.01 (−0.01, 0.04)	−0.01 (−0.06, 0.03)	0.49 (0.11)
	Women	0.01 (−0.03, 0.06)	0.02 (−0.01, 0.04)	0.01 (−0.03. 0.04)	0.59 (0.57)
∆LDL-C ^‡^	All	−0.48 (−0.55, −0.41)	−0.44 (−0.48, −0.40)	−0.54 (−0.60, −0.48)	0.047 (0.02)
	Men	−0.66 (−0.77, −0.55)	−0.59 (−0.65, −0.53)	−0.70 (−0.81, −0.60)	0.23 (0.31)
	Women	−0.37 (−0.46, −0.27)	−0.35 (−0.40, −0.30)	−0.43 (−0.51, −0.35)	0.14 (0.06)

* In parentheses: results excluding individuals reporting dietary changes in the past and misreporters of energy (*n* = 2001 remained); ^†^ Analyses with blood lipids at follow-up were adjusted for the following variables collected at baseline: sex, season, total energy intake, smoking, education, leisure-time physical activity, alcohol habits, waist circumference, and follow-up time; ^‡^ Analyses with change in blood lipids during follow-up were also adjusted for ln-transformed baseline lipid concentrations.

**Table 3:** Odds ratio (95% CI) of developing dyslipidemia and other cardiometabolic risk factors according to the diet quality index.

Traits	Sample Size (Incident Cases)	Incident Cases in Low/Medium/High Index Groups	Low (0–1)	Medium (2–4)	High (5–6)	*p*-Trend *
High triglycerides ^†^	2587 (168)	11%/6%/6%	1.00	0.57 (0.37–0.88)	0.54 (0.31–0.95)	0.02 (0.02)
Low HDL-C ^†^	2330 (380)	16%/17%/17%	1.00	1.03 (0.71–1.48)	1.07 (0.69–1.65)	0.93 (0.20)
High LDL-C ^†^	1686 (876)	54%/53%/48%	1.00	0.94 (0.69–1.28)	0.75 (0.51–1.09)	0.03 (0.03)
Elevated waist circumference ^†^	2779 (938)	37%/33%/35%	1.00	0.85 (0.66–1.09)	0.92 (0.68–1.25)	0.38 (0.08)
Hypertension ^†^	746 (485)	56%/66%/66%	1.00	1.77 (1.10–2.84)	1.85 (1.02–3.34)	0.22 (0.29)
Elevated plasma glucose ^†^	1885 (1043)	52%/56%/57%	1.00	1.14 (0.85–1.54)	1.29 (0.91–1.85)	0.02 (0.13)

Logistic regression was used to estimate OR (95% CI) and adjusted for age, sex, season, total energy intake, smoking, education, leisure-time physical activity, alcohol habits, waist circumference; * *p*-Values in parentheses: results excluding individuals reporting dietary changes in the past and misreporters of energy; ^†^ The following definitions were used: high triglycerides: ≥1.7 mmol/L and/or triglyceride lowering treatment; low HDL-C: <1.0 mmol/L for men and <1.3 mmol/L for women; high LDL-C: >4.1 mmol/L and/or lipid lowering treatment; elevated waist circumference: ≥102 cm for men and ≥88 for women; hypertension: ≥130 mmHg SBP or ≥85 mmHg DBP or antihypertensive drug treatment; elevated plasma glucose; ≥5.6 mmol/L or glucose lowering drug treatment.

**Table 4:** Blood lipids at baseline and change in blood lipids during follow-up (mean and 95% CI) by categories of diet index in strata of genetic risk scores.

Traits	GRS Groups *	Diet Quality Index			*p*-Trend	*p*-Interaction ^†^
		Low	Medium	High		
TG ^‡^	Low GRS_TG_	1.24 (1.17, 1.32)	1.20 (1.16, 1.24)	1.14 (1.08, 1.21)	0.07 (0.01)	0.46 (0.55)
	High GRS_TG_	1.38 (1.29, 1.47)	1.33 (1.28, 1.37)	1.38 (1.30, 1.45)	0.97 (0.41)	
HDL-C ^‡^	Low GRS_HDL_	1.43 (1.39, 1.48)	1.45 (1.43, 1.48)	1.45 (1.41, 1.49)	0.26 (0.27)	0.99 (0.66)
	High GRS_HDL_	1.31 (1.26, 1.36)	1.35 (1.26, 1.36)	1.38 (1.34, 1.42)	0.04 (0.001)	
LDL-C ^‡^	Low GRS_LDL_	4.04 (3.90, 4.18)	3.89 (3.81, 3.96)	3.83 (3.80, 4.05)	0.16 (0.09)	0.20 (0.52)
	High GRS_LDL_	4.28 (4.13, 4.43)	4.30 (4.23, 4.37)	4.37 (4.25, 4.49)	0.10 (0.77)	
∆-TG ^§^	Low GRS_TG_	−0.12 (−0.18, −0.06)	−0.15 (−0.18, −0.12)	−0.12 (−0.17, −0.06)	0.88 (0.43)	0.19 (0.94)
	High GRS_TG_	−0.05 (−0.12, 0.03)	−0.12 (−0.16, −0.08)	−0.10 (−0.17, −0.04)	0.06 (0.03)	
∆-HDL-C ^§^	Low GRS_HDL_	−0.04 (−0.09, 0.001)	0.02 (−0.002, 0.05)	0.01 (−0.03, 0.05)	0.05 (0.02)	0.04 (0.06)
	High GRS_HDL_	0.03 (−0.01, 0.08)	0.01 (−0.02, 0.03)	−0.01 (−0.05, 0.03)	0.35 (0.66)	
∆-LDL-C ^§^	Low GRS_LDL_	−0.39 (−0.48, −0.30)	−0.36 (−0.41, −0.31)	−0.45 (−0.53, −0.36)	0.15 (0.15)	0.33 (0.29)
	High GRS_LDL_	−0.59 (−0.70, −0.47)	−0.53 (−0.59, −0.48)	−0.64 (−0.73, −0.56)	0.16 (0.07)	

Abbreviations: GRS, genetic risk score; TG, triglycerides; * The genetic risk scores were split by the median value; ^†^ The interactions were examined with continuous variables of the diet categories and the genetic risk scores; *p*-Values in parentheses: results excluding individuals reporting dietary changes in the past and misreporters of energy (*n* = 1966 remained); ^‡^ Baseline lipids were adjusted for age, sex, season, total energy intake, waist circumference, smoking, alcohol habits, leisure-time physical activity, and education; ^§^ Change in blood lipids was adjusted for sex, age, follow-up time, baseline bloodä lipid concentrations, season, total energy intake, education, smoking, leisure-time physical activity, alcohol consumption and waist circumference.

## Supplementary Materials

The following are available online at http://www.mdpi.com/2072-6643/8/5/274/s1, Table S1: Characteristics of the included single nucleotide polymorphisms; Table S2: Characteristics of individuals from the baseline examination (1991–1994) that either attended the re-examination (2007–2012) or did not attend the re-examination because of death or other reasons; Table S3: Baseline blood lipids and lipoprotein subfractions in categories of the diet quality index in the Malmö Diet and Cancer cohort; Table S4: Odds ratio (95% CI) of prevalent dyslipidemia and other cardiometabolic risk factors according to the diet quality index; Table S5: Blood lipid concentrations at baseline and follow-up (mean and 95% CI) and change in blood lipids during follow-up (mean change and 95% CI) by adherence to specific components; Table S6: Association (only *p*-values are shown) between the specific dietary components and blood lipid concentrations at baseline and follow-up and change in blood lipids during follow-up in men and women in the Malmö Diet and Cancer cohort.
