# Supplementary material for: Diet Quality and Change in Blood Lipids during 16 Years of Follow-up and Their Interaction with Genetic Risk for Dyslipidemia
Source: Nutrients. 2016 May 9;8(5):274. doi: 10.3390/nu8050274 (PMC4882687; doi:10.3390/nu8050274)
Supplement: Supplementary file 1 [file nutrients-08-00274-s001.doc]

Supplemental Materials: Diet Quality and Change in Blood Lipids during 16 Years of Follow-up and Their Interaction with Genetic Risk for Dyslipidemia

Emily Sonestedt, Sophie Hellstrand, Isabel Drake, Christina-Alexandra Schulz,
Ulrika Ericson, Joanna Hlebowicz, Margaretha M. Persson, Bo Gullberg, Bo Hedblad,
Gunnar Engström and Marju Orho-Melander

**Table S1.** Characteristics of the included single nucleotide polymorphisms.

| **Chromosome** | **Locus** | **SNP** | **Alleles */MAF** | **Lead Trait/Additional Traits (Effect Size)** |
| --- | --- | --- | --- | --- |
| 1 | *LDLRAP1* | rs12027135 | T/A/0.45 | LDL-C (−1.10) |
| 1 | *PABPC4* | rs4660293 | A/G/0.25 | HDL-C (−0.48) |
| 1 | *PCSK9* | rs2479409 | A/G/0.35 | LDL-C (2.01) |
| 1 | *SORT1* | rs629301 | A/C/0.23 | LDL-C (−5.56) |
| 1 | *ZNF648* | rs1689800 | T/C/0.34 | HDL-C (−0.47) |
| 1 | *GALNT2* | rs4846914 | A/G/0.40 | HDL-C (−0.61)/TG (2.76) |
| 1 | *IRF2BP2* | rs514230 | A/T/0.48 | LDL-C (−1.13) |
| 2 | *APOB* | rs1042034 | A/G/0.20 | TG (−5.99)/HDL-C (0.90) |
| 2 | *APOB* | rs1367117 | G/A/0.34 | LDL-C (4.05) |
| 2 | *GCKR* | rs1260326 | C/T/0.37 | TG (8.76) |
| 2 | *ABCG5/8* | rs4299376 | T/G/0.29 | LDL-C (2.75) |
| 2 | *COBLL1* | rs12328675 | T/C/0.12 | HDL-C (0.68) |
| 2 | *IRS1* | rs2972146 | A/C/0.37 | HDL-C (0.46)/TG (−1.89) |
| 3 | *MSL2L1* | rs645040 | T/G/0.22 | TG (−2.22) |
| 4 | *KLHL8* | rs442177 | A/C/0.43 | TG (−2.25) |
| 4 | *SLC39A8* | rs13107325 | C/T/0.05 | HDL-C (−0.84) |
| 5 | *ARL15* | rs6450176 | G/A/0.25 | HDL-C (−0.49) |
| 5 | *MAP3K1* | rs9686661 | C/T/0.16 | TG (2.57) |
| 5 | *HMGCR* | rs12916 | T/C/0.41 | LDL-C (2.45) |
| 5 | *TIMD4* | rs6882076 | C/T/0.36 | LDL-C (−1.67)/TG (−2.63) |
| 6 | *MYLIP* | rs3757354 | G/A/0.24 | LDL-C (−1.43) |
| 6 | *HFE* | rs1800562 | G/A/0.05 | LDL-C (−2.22) |
| 6 | *HLA* | rs3177928 | G/A/0.13 | LDL-C (1.83) |
| 6 | *HLA* | rs2247056 | G/A/0.27 | TG (−2.99) |
| 6 | *C6orf106* | rs2814944 | G/A/0.16 | HDL-C (−0.49) |
| 6 | *FRK* | rs9488822 | A/T/0.31 | LDL-C (−0.89) |
| 6 | *CITED2* | rs605066 | T/C/0.44 | HDL-C (−0.39) |
| 6 | *LPA* | rs1564348 | A/G/0.15 | LDL-C (1.95) |
| 7 | *DNAH11* | rs12670798 | T/C/0.25 | LDL-C (1.26) |
| 7 | *MLXIPL* | rs17145738 | C/T/0.12 | TG (−9.32)/HDL-C (0.57) |
| 8 | *PPP1R3B* | rs9987289 | G/A/0.10 | HDL-C (−1.21)/LDL-C (−2.22) |
| 8 | *PINX1* | rs11776767 | G/C/0.34 | TG (2.01) |
| 8 | *NAT2* | rs1495741 | A/G/0.22 | TG (2.85) |
| 8 | *LPL* | rs12678919 | A/G/0.09 | TG (−13.64)/HDL-C (2.25) |
| 8 | *CYP7A1* | rs2081687 | C/T/0.34 | LDL-C (0.95) |
| 8 | *TRPS1* | rs2293889 | G/T/0.43 | HDL-C (−0.44) |
| 8 | *TRIB1* | rs2954029 | A/T/0.47 | TG (−5.64)/LDL-C (−1.84)/HDL-C (0.61) |
| 9 | *TTC39B* | rs581080 | C/G/0.19 | HDL-C (−0.65) |
| 9 | *ABCA1* | rs1883025 | G/A/0.24 | HDL-C (−0.94) |
| 9 | *ABO* | rs9411489 | G/A/0.21 | LDL-C (2.24) |
| 10 | *CYP26A1* | rs2068888 | G/A/0.44 | TG (−2.28) |
| 10 | *GPAM* | rs2255141 | G/A/0.29 | LDL-C (1.08) |
| 11 | *AMPD3* | rs2923084 | A/G/0.17 | HDL-C (−0.41) |
| 11 | *LRP4* | rs3136441 | T/C/0.14 | HDL-C (0.78) |
| 11 | *FADS123* | rs174546 | C/T/0.33 | TG (3.82)/HDL-C (−0.73)/LDL-C (−1.71) |
| 11 | *APOA1* | rs964184 | C/G/0.13 | TG (16.95)/HDL-C (−1.50)/LDL-C (2.85) |
| 11 | *UBASH3B* | rs7941030 | T/C/0.39 | HDL-C (0.31) |
| 11 | *ST3GAL4* | rs11220462 | C/T/0.14 | LDL-C (1.95) |
| 12 | *PDE3A* | rs7134375 | C/A/0.43 | HDL-C (0.40) |
| 12 | *LRP1* | rs11613352 | C/T/0.27 | TG (−2.70)/HDL-C (0.46) |
| 12 | *MVK* | rs7134594 | T/C/0.46 | HDL-C (−0.44) |
| 12 | *BRAP* | rs11065987 | A/G/0.42 | LDL-C (−0.97) |
| 12 | *HNF1A* | rs1169288 | T/G/0.32 | LDL-C (1.42) |
| 12 | *SBNO1* | rs4759375 | C/T/0.09 | HDL-C (0.86) |
| 12 | *ZNF664* | rs4765127 | G/T/0.33 | HDL-C (0.44)/TG (−2.42) |
| 14 | *NYNRIN* | rs8017377 | G/A/0.46 | LDL-C (1.14) |
| 15 | *CAPN3* | rs2412710 | G/A/0.02 | TG (7.00) |
| 15 | *FRMD5* | rs2929282 | A/T/0.04 | TG (5.13) |
| 15 | *LIPC* | rs1532085 | G/A/0.38 | HDL-C (1.45)/TG (2.99) |
| 15 | *LACTB* | rs2652834 | C/T/0.21 | HDL-C (−0.39) |
| 16 | *CTF1* | rs11649653 | C/G/0.41 | TG (−2.13) |
| 16 | *CETP* | rs3764261 | G/T/0.33 | HDL-C (3.39)/LDL-C (−1.45)/TG (−2.88) |
| 16 | *LCAT* | rs16942887 | G/A/0.14 | HDL-C (1.27) |
| 16 | *HPR* | rs2000999 | G/A/0.22 | LDL-C (2.00) |
| 16 | *CMIP* | rs2925979 | G/A/0.30 | HDL-C (−0.45) |
| 17 | *STARD3* | rs11869286 | C/G/0.32 | HDL-C (−0.48) |
| 17 | *PGS1* | rs4129767 | T/C/0.48 | HDL-C (−0.39) |
| 18 | *LIPG* | rs7241918 | T/G/0.17 | HDL-C (−1.31) |
| 18 | *MC4R* | rs12967135 | G/A/0.23 | HDL-C (−0.42) |
| 19 | *ANGPTL4* | rs7255436 | A/C/0.44 | HDL-C (−0.45) |
| 19 | *LDLR* | rs6511720 | G/T/0.10 | LDL-C (−6.99) |
| 19 | *LOC55908* | rs737337 | T/C/0.11 | HDL-C (−0.64) |
| 19 | *CILP2* | rs10401969 | T/C/0.10 | TG (−7.83)/LDL-C (−3.11) |
| 19 | *APOE* | rs439401 | C/T/0.36 | TG (−5.50) |
| 19 | *APOE* | rs4420638 | A/G/0.20 | LDL-C (7.14)/HDL-C (−1.06) |
| 20 | *MAFB* | rs2902940 | A/G/0.28 | LDL-C (−0.98) |
| 20 | *TOP1* | rs6029526 | T/A/0.48 | LDL-C (1.39) |
| 20 | *HNF4A* | rs1800961 | C/T/0.04 | HDL-C (−1.88) |
| 22 | *UBE2L3* | rs181362 | G/A/0.22 | HDL-C (−0.46) |
| 22 | *PLA2G6* | rs5756931 | T/C/0.36 | TG (−1.54) |

* The risk allele is marked. For a few of the SNPs risk allele are different depending on the trait (PP1R3B [HDL-C:A; LDL-C:G], FADS123 [TG, HDL-C:T; LDL-C:C], LIPC [HDL-C:G; TG:A]); however the risk allele was defined as the association with the lead trait.

**Table S2.** Characteristics of individuals from the baseline examination (1991–1994) that either attended the re-examination (2007–2012) or did not attend the re-examination because of death or other reasons.

|  | **Men** | | | **Women** | | |
| --- | --- | --- | --- | --- | --- | --- |
|  | **Attendees  (*n* = 1522)** | **Died before Start of Re-Examination (*n* = 551)** | **Non-Attending Due to Unwillingness, Sickness, Emigration, Lack of Information in Registers (*n* = 499)** | **Attendees  (*n* = 2212)** | **Died before Start of Re-Examination (*n* = 485)** | **Non-Attending Due to Unwillingness, Sickness, Emigration, Lack of Information in Registers (*n* = 834)** |
| Age, y | 56.3 (5.8) | 60.4 (5.3) * | 58.1 (6.0) * | 56.3 (5.6) | 60.5 (5.4) * | 58.7 (6.1) * |
| Waist circumference, cm | 92.3 (9.3) | 95.2 (11.4) * | 95.3 (10.7) * | 76.7 (9.5) | 79.7 (11.3) * | 79.2 (11.7) * |
| Diet score | 3.00 (1.36) | 2.79 (1.31) * | 2.85 (1.38) * | 3.18 (1.44) | 3.00 (1.83) * | 2.99 (1.45) * |
| Saturated fat, E% | 16.1 (3.9) | 16.3 (4.1) | 16.1 (3.8) | 15.6 (3.7) | 16.0 (4.1) | 15.8 (4.0) |
| Polyunsaturated fat, E% | 6.27 (1.58) | 6.28 (1.62) | 6.18 (1.68) | 5.89 (1.50) | 5.79 (1.47) | 5.74 (1.48) * |
| Sucrose, E% | 7.85 (3.31) | 8.11 (4.00) | 7.99 (3.92) | 8.67 (3.10) | 8.59 (3.65) | 8.93 (3.49) |
| Fiber, g/MJ | 2.14 (0.63) | 2.01 (0.57) * | 2.11 (0.64) | 2.44 (0.68) | 2.32 (0.69) * | 2.41 (0.73) |
| Fruit and vegetables, g/day | 365 (178) | 333 (183) * | 374 (195) | 423 (183) | 391 (190) * | 406 (185) * |
| Fish and shellfish, g/week | 342 (276) | 351 (276) | 352 (283) | 312 (215) | 312 (214) | 300 (219) |
| Triglycerides, mmol/ | 1.48 (0.82) | 1.60 (0.87) * | 1.64 (1.18) * | 1.19 (0.61) | 1.41 (0.79) * | 1.39 (0.82) * |
| HDL-C, mmol/L | 1.22 (0.30) | 1.17 (0.32) * | 1.19 (0.30) | 1.53 (0.36) | 1.44 (0.39) * | 1.46 (0.37) * |
| LDL-C, mmol/L | 4.14 (0.88) | 4.08 (0.95) | 4.08 (0.89) | 4.13 (1.04) | 4.33 (1.09) * | 4.32 (1.02) * |
| GRS-TG, *z*-score | −0.04 (1.00) | −0.08 (1.02) | 0.07 (1.03) * | −0.001 (1.02) | 0.09 (0.98) * | 0.01 (1.02) |
| GRS-HDLC, *z*-score | −0.002 (1.00) | 0.02 (0.98) | 0.02 (0.96) | 0.02 (0.99) | 0.11 (0.99) | −0.04 (1.03) |
| GRS-LDLC, *z*-score | 0.003 (0.97) | −0.02 (0.97) | 0.09 (1.02) | −0.01 (0.99) | 0.08 (1.05) | 0.04 (1.02) |
| Smokers, % | 24.1% | 42.5% * | 32.4% * | 23.0% | 39.4% * | 30.8% * |
| Zero-consumers of alcohol, % | 2.9% | 7.3% * | 6.0% * | 4.9% | 14.1% * | 10.3% * |
| High-consumers of alcohol, % | 18.5% | 20.0% | 20.3% | 16.4% | 13.2% | 9.7% * |
| High physical activity, % | 20.0% | 20.2% | 19.8% | 19.8% | 20.1% | 22.8% |
| Past food habit change, % | 21.7% | 28.3% * | 22.1% | 26.9% | 30.1% | 30.2% |
| Misreporters of energy, % | 17.2% | 16.1% | 14.9% | 21.2% | 22.7% | 20.5% |
| Lipid-lowering drugs, % | 2.4% | 4.0% * | 3.4% | 1.5% | 2.7% | 2.3% |

* Statistically significant difference (*p* > 0.05) compared with attendees. Number of individuals with information on different variables: age, 2572 men/3531 women; waist, 2565/3529; dietary factors, 2403/3377; triglycerides, 2282/3228; HDL-C, 2260/3193; LDL-C, 2217/3154; GRS, 2526/3456; smoking habits, 2411/3377; alcohol habits, 2403/3377; physical activity, 2196/3220; past food habit change, 2409/3376; misreporters, 2389/3363; lipid-lowering drugs; 2572/3531.

**Table S3.** Baseline blood lipids and lipoprotein subfractions in categories of the diet quality index in the Malmö Diet and Cancer cohort.

|  | **Low (0–1)  *n* = 379** | **Medium (2–4)  *n* = 2221** | **High (5–6)  *n* = 552** | ***p*-Trend *** |
| --- | --- | --- | --- | --- |
|  | Mean (SE) | | |  |
| Total cholesterol (mmol/L) | 6.12 (0.06) | 6.08 (0.03) | 6.15 (0.05) | 0.38 (0.98) |
| Triglycerides (mmol/L) | 1.31 (0.03) | 1.26 (0.02) | 1.26 (0.03) | 0.23 (0.04) |
| HDL-C (mmol/L) | 1.37 (0.02) | 1.40 (0.01) | 1.41 (0.02) | 0.02 (0.004) |
| LDL-C (mmol/L) | 4.15 (0.05) | 4.10 (0.03) | 4.16 (0.05) | 0.74 (0.57) |
| HDL-S (nmol/L) | 2722 (100) | 2828 (54) | 2905 (86) | 0.09 (0.19) |
| HDL-L (nmol/L) | 1633 (59) | 1669 (32) | 1702 (50) | 0.17 (0.15) |
| LDL-VS (nmol/L) | 107 (3) | 102 (2) | 102 (3) | 0.58 (0.50) |
| LDL-S (nmol/L) | 80.7 (3.6) | 73.1 (1.9) | 72.5 (3.1) | 0.11 (0.64) |
| LDL-M (nmol/L) | 123 (4) | 116 (2) | 114 (4) | 0.09 (0.48) |
| LDL-L (nmol/L) | 436 (10) | 427 (5) | 411 (8) | 0.046 (0.15) |
| IDL-S (nmol/L) | 119 (3) | 116 (1) | 115 (2) | 0.36 (0.74) |
| IDL-L (nmol/L) | 217 (6) | 218 (3) | 214 (5) | 0.98 (0.60) |
| VLDL-S (nmol/L) | 53.4 (1.2) | 52.1 (0.6) | 52.0 (1.0) | 0.48 (0.74) |
| VLDL-M (nmol/L) | 36.7 (0.9) | 34.9 (0.5) | 34.2 (0.7) | 0.04 (0.11) |
| VLDL-L (nmol/L) | 9.62 (0.29) | 9.01 (0.15) | 8.78 (0.25) | 0.01 (0.01) |
| LDL peak diameter (nm) | 220.1 (0.3) | 220.6 (0.2) | 220.7 (0.3) | 0.59 (0.67) |
|  | OR (95% CI) | | |  |
| High triglycerides ^†^ | 20.3% | 17.6% | 17.8% |  |
| OR (95% CI) | 1.00 | 0.82 (0.61, 1.11) | 0.98 (0.69, 1.41) | 0.69 (0.48) |
| Low HDL-C ^†^ | 34.0% | 25.5% | 22.8% |  |
| OR (95% CI) | 1.00 | 0.73 (0.57, 0.94) | 0.68 (0.50, 0.93) | **0.009 (0.001)** |
| High LDL-C ^†^ | 46.7% | 46.6% | 46.0% |  |
| OR (95% CI) | 1.00 | 0.95 (0.76, 1.19) | 0.94 (0.72, 1.24) | 0.86 (0.59) |

* *p*-Values in parenthesis: results excluding individuals reporting dietary changes in the past and misreporters of energy; ^†^ The following definitions were used: high triglycerides: ≥1.7 mmol/L and/or triglyceride lowering treatment; low HDL-C: <1.0 mmol/L for men and <1.3 mmol/L for women; high LDL-C: >4.1 mmol/L and/or lipid lowering treatment.

**Table S4.** Odds ratio (95% CI) of dyslipidemia and other cardiometabolic risk factors at baseline according to the diet quality index and sex.

|  | **Men** | | | | **Women** | | | |
| --- | --- | --- | --- | --- | --- | --- | --- | --- |
|  | **Low  (0–1)** | **Medium  (2–4)** | **High  (5–6)** | ***p*-Trend *** | **Low (0–1)** | **Medium  (2–4)** | **High  (5–6)** | ***p*-Trend *** |
| High triglycerides ^†^ | 27.3% | 24.3% | 25.3% |  | 15.7% | 13.0% | 14.3% |  |
| OR (95% CI) | 1.00 | 0.82  (0.54, 1.25) | 1.00  (0.59, 1.72) | 0.65 (0.56) | 1.00 | 0.82  (0.54, 1.24) | 0.97  (0.59, 1.60) | 0.84 (0.09) |
| Low HDL-C ^†^ | 34.0% | 23.5% | 23.6% |  | 34.1% | 26.9% | 22.5% |  |
| OR (95% CI) | 1.00 | **0.66  (0.44, 0.98)** | 0.75  (0.44, 1.26) | 0.47 (0.08) | 1.00 | 0.77  (0.56, 1.07) | **0.65  (0.44, 0.97)** | **0.006 (0.004)** |
| High LDL-C ^†^ | 48.0% | 49.4% | 46.6% |  | 45.9% | 44.7% | 45.8% |  |
| OR (95% CI) | 1.00 | 0.99  (0.70, 1.42) | 0.87  (0.55, 1.37) | 0.45 (0.38) | 1.00 | 0.92  (0.68, 1.24) | 0.97  (0.68, 1.38) | 0.39 (0.89) |
| Elevated waist circumference ^†^ | 15.3% | 12.5% | 12.1% |  | 12.7% | 11.0% | 10.3% |  |
| OR (95% CI) | 1.00 | 0.77  (0.46–1.28) | 0.83  (0.42–1.62) | 0.84 (0.87) | 1.00 | 0.92  (0.60–1.43) | 0.85  (0.50–1.45) | 0.76 (0.86) |
| Hypertension ^†^ | 82.0% | 81.8% | 80.5% |  | 69.4% | 72.6% | 75.9% |  |
| OR (95% CI) | 1.00 | 0.85  (0.53–1.38) | 0.74  (0.41–1.35) | 0.51 (0.60) | 1.00 | 1.11  (0.80–1.54) | 1.32  (0.89–1.97) | 0.11 (0.42) |
| Elevated plasma glucose ^†^ | 55.7% | 54.2% | 46.6% |  | 34.5% | 31.3% | 30.6% |  |
| OR (95% CI) | 1.00 | 0.82  (0.57–1.19) | 0.63  (0.39–1.00) | 0.06 (0.09) | 1.00 | 0.82  (0.60–1.12) | 0.82  (0.56–1.19) | 0.43 (0.44) |

Logistic regression was used to estimate OR (95% CI) and adjusted for age, season, total energy intake, smoking, education, leisure-time physical activity, alcohol habits, waist circumference. * P-values in parenthesis: results excluding individuals reporting dietary changes in the past and misreporters of energy; ^†^ The following definitions were used: high triglycerides: ≥1.7 mmol/L and/or triglyceride lowering treatment; low HDL-C: <1.0 mmol/L for men and <1.3 mmol/L for women; high LDL-C: >4.1 mmol/L and/or lipid lowering treatment; elevated waist circumference: ≥102 cm for men and ≥88 for women; hypertension: ≥130 mm Hg SBP or ≥85 mm Hg DBP or antihypertensive drug treatment; elevated plasma glucose; ≥5.6 mmol/L or glucose lowering drug treatment.

**Table S5.** Blood lipid concentrations at baseline and follow-up (mean and 95% CI) and change in blood lipids during follow-up (mean change and 95% CI) by adherence to specific components.

|  |  | **Triglycerides** | | | **HDL-C** | | | **LDL-C** | | |
| --- | --- | --- | --- | --- | --- | --- | --- | --- | --- | --- |
|  |  | **Baseline *** | **Follow-up ^†^** | **Change ^‡^** | **Baseline *** | **Follow-up ^†^** | **Change ^‡^** | **Baseline *** | **Follow-up ^†^** | **Change ^‡^** |
| Saturated fat | Non-adherence | 1.26  (1.23, 1.29) | 1.12  (1.10, 1.15) | −0.13  (−0.15, −0.10) | 1.40  (1.39, 1.42) | 1.41  (1.39, 1.43) | 0.002  (−0.02, 0.02) | 4.12  (4.06, 4.17) | 3.66  (3.61, 3.70) | −0.45  (−0.49, −0.53) |
|  | Adherence ^§^ | 1.28  (1.24, 1.32) | 1.14  (1.11, 1.18) | −0.12  (−0.15, −0.09) | 1.39  (1.37, 1.41) | 1.41  (1.39, 1.44) | 0.02  (−0.003, 0.04) | 4.13  (4.06, 4.20) | 3.65  (3.59, 3.70) | −0.48  (−0.53, −0.44) |
|  | *p*-value ^††^ | 0.38  (0.38) | 0.40  (0.65) | 0.75  (0.92) | 0.29  (0.60) | 0.92  (0.77) | 0.15 (0.28) | 0.86  (0.17) | 0.61  (0.11) | 0.26  (0.26) |
| Polyunsaturated fat | Non-adherence | 1.26  (1.22, 1.31) | 1.12  (1.08, 1.16) | −0.13  (−0.16, −0.10) | 1.40  (1.37, 1.42) | 1.41  (1.39, 1.44) | 0.01  (−0.01, 0.04) | 4.16  (4.09, 4.23) | 3.68  (3.62, 3.74) | −0.46  (−0.51, −0.41) |
|  | Adherence ^§^ | 1.27  (1.24, 1.30) | 1.14  (1.11, 1.16) | −0.12  (−0.15, −0.10) | 1.40  (1.38, 1.42) | 1.41  (1.39, 1.43) | 0.004  (−0.01, 0.02) | 4.10  (4.05, 4.16) | 3.64  (3.60, 3.69) | −0.47  (−0.51, −0.43) |
|  | *p*-value ^††^ | 0.96  (0.20) | 0.81  (0.53) | 0.63  (0.85) | 0.69  (0.58) | 0.52  (0.54) | 0.44  (0.55) | 0.14  (**0.02**) | 0.15  (**0.03**) | 0.72  (0.54) |
| Sucrose | Non-adherence | 1.30  (1.26, 1.35) | 1.15  (1.11, 1.19) | −0.12  (−0.16, −0.09) | 1.35  (1.33, 1.37) | 1.36  (1.33, 1.39) | −0.01  (−0.03, 0.02) | 4.09  (4.01, 4.16) | 3.65  (3.59, 3.71) | −0.45  (−0.51, −0.40) |
|  | Adherence ^§^ | 1.25  (1.22, 1.28) | 1.12  (1.10, 1.15) | −0.12  (−0.15, −0.10) | 1.42  (1.40, 1.43) | 1.43  (1.41, 1.45) | 0.01  (−0.01, 0.03) | 4.13  (4.08, 4.19) | 3.66  (3.61, 3.70) | −0.47  (−0.51, −0.43) |
|  | *p*-value ^††^ | **0.004  (0.04)** | 0.18  (0.32) | 0.77  (0.56) | **3 × 10^−7^  (6 × 10^−6^)** | **1 × 10^−4^  (2 × 10^−4^)** | 0.16  (0.15) | 0.24  (0.32) | 0.55  (0.76) | 0.59  (0.59) |
| Fiber | Non-adherence | 1.27  (1.23, 1.30) | 1.15  (1.12, 1.18) | −0.11  (−0.13, −0.08) | 1.40  (1.38, 1.42) | 1.41  (1.38, 1.43) | 0.003  (−0.02, 0.02) | 4.12  (4.07, 4.18) | 3.67  (3.62, 3.72) | −0.45  (−0.49, −0.41) |
|  | Adherence ^§^ | 1.27  (1.23, 1.31) | 1.10  (1.07, 1.14) | −0.15  (−0.18, −0.12) | 1.40  (1.38, 1.42) | 1.42  (1.39, 1.45) | 0.01  (−0.01, 0.03) | 4.11  (4.05, 4.18) | 3.63  (3.58, 3.69) | −0.49  (−0.53, −0.44) |
|  | *p*-value ^††^ | 0.80  (0.14) | **0.04  (4 × 10^−4^)** | **0.007  (0.002)** | 0.63  (0.42) | 0.39  (0.21) | 0.33  (0.23) | 0.64  (0.83) | 0.23  (0.14) | 0.14  (0.08) |
| Fruit and vegetables | Non-adherence | 1.27  (1.23, 1.30) | 1.14  (1.11, 1.17) | −0.11  (−0.14, −0.09) | 1.40  (1.38, 1.42) | 1.41  (1,39, 1.44) | 0.01  (−0.01, 0.03) | 4.11  (4.05, 4.17) | 3.66  (3.62, 3.71) | −0.45  (−0.49, −0.40) |
|  | Adherence ^§^ | 1.27  (1.23, 1.30) | 1.12  (1.09, 1.15) | −0.14  (−0.17, −0.11) | 1.40  (1.38, 1.42) | 1.41  (1.38, 1.43) | 0.001  (−0.02, 0.02) | 4.13  (4.07, 4.20) | 3.64  (3.59, 3.70) | −0.49  (−0.53, −0.44) |
|  | *p*-value ^††^ | 0.88  (0.17) | 0.22  (0.06) | 0.17  (0.20) | 0.86  (0.25) | 0.76  (0.38) | 0.36  (0.84) | 0.70  (0.84) | 0.42  (0.24) | 0.09  (0.20) |
| Fish and shellfish | Non-adherence | 1.28  (1.25, 1.31) | 1.14  (1.11, 1.17) | −0.12  (−0.14, −0.09) | 1.39  (1.37, 1.41) | 1.40  (1.38, 1.42) | 0.01  (−0.01, 0.02) | 4.10  (4.04, 4.16) | 3.65  (3.60, 3.70) | −0.46  (−0.50, −0.42) |
|  | Adherence ^§^ | 1.25  (1.22, 1.29) | 1.12  (1.08, 1.15) | −0.13  (−0.16, −0.10) | 1.41  (1.39, 1.43) | 1.42  (1.40, 1.45) | 0.01  (−0.01, 0.03) | 4.14  (4.08, 4.21) | 3.66  (3.61, 3.71) | −0.47  (−0.52, −0.43) |
|  | *p*-value ^††^ | 0.08  (0.22) | 0.10  (0.19) | 0.30  (0.27) | 0.06  (0.07) | 0.23  (0.23) | 0.75  (0.90) | 0.14  (0.21) | 0.77  (0.82) | 0.53  (0.23) |

* Baseline associations were adjusted for age, sex, season, total energy intake, smoking, education, leisure-time physical activity, alcohol habits, and waist circumference; ^†^ Follow-up associations were also adjusted for follow-up time; ^‡^ Change in standard blood lipids was also adjusted for baseline lipid concentrations and follow-up time; ^§^ *p*-values in parenthesis: results excluding individuals reporting dietary changes in the past and misreporters of energy (*n* = 2001 remained); ^††^ Adherence was defined as: saturated fat ≤14 E%, polyunsaturated fat 5–10 E%, sucrose ≤10 E%, fiber ≥2.4 g/MJ, fruit and vegetables ≥400 g/day and fish and shellfish ≥300 g/week.

**Table S6.** Association (only *p*-values are shown *) between the specific dietary components and standard blood lipid concentrations at baseline and follow-up and change in standard blood lipids during follow-up in men and women in the Malmö Diet and Cancer cohort.

|  |  | **Triglycerides** | | | **HDL-C** | | | **LDL-C** | | |
| --- | --- | --- | --- | --- | --- | --- | --- | --- | --- | --- |
|  |  | **Baseline** | **Follow-up** | **Change** | **Baseline** | **Follow-up** | **Change** | **Baseline** | **Follow-up** | **Change** |
| Saturated fat | Men | 0.15 (0.50) | 0.40 (0.88) | 0.61 (0.98) | 0.15 (0.34) | 0.50 (0.43) | 0.46 (0.92) | 0.31 (**0.04**) | 0.61 (0.27) | 0.87 (0.85) |
|  | Women | 0.90 (0.07) | 0.90 (0.35) | 0.89 (0.83) | 0.74 (0.88) | 0.47 (0.23) | 0.14 (0.15) | 0.80 (0.53) | 0.79 (0.21) | 0.25 (0.16) |
| Polyunsaturated fat | Men | 0.70 (0.20) | 0.93 (0.76) | 0.90 (0.13) | 0.17 (0.22) | 0.73 (0.16) | 0.77 (0.28) | 0.07 (0.09) | 0.07 (**0.02**) | 0.40 (0.14) |
|  | Women | 0.79 (0.62) | 0.80 (0.33) | 0.67 (0.28) | 0.72 (0.88) | 0.36 (0.84) | 0.44 (0.98) | 0.54 (0.09) | 0.61 (0.37) | 0.86 (0.65) |
| Sucrose | Men | **0.002 (0.045)** | 0.22 (0.22) | 0.88 (0.68) | **0.003 (0.004)** | **0.02 (0.002)** | 0.32 (0.07) | 0.25 (0.18) | 0.65 (0.82) | 0.28 (0.56) |
|  | Women | 0.13 (0.19) | 0.33 (0.62) | 0.47 (0.56) | **<0.001 (<0.001)** | **0.003 (0.02)** | 0.32 (0.62) | **0.03 (0.03)** | 0.69 (0.57) | 0.15 (0.25) |
| Fiber | Men | 0.93 (0.23) | 0.21 (**0.02**) | 0.07 (**0.04**) | 0.59 (0.93) | 0.82 (0.85) | 0.66 (0.72) | 0.22 (0.16) | 0.11 (0.09) | 0.23 (0.38) |
|  | Women | 0.70 (0.34) | 0.08 (**0.01**) | 0.03 (**0.02**) | 0.43 (0.37) | 0.25 (0.17) | 0.33 (0.23) | 0.89 (0.74) | 0.67 (0.44) | 0.32 (0.15) |
| Fruit and vegetables | Men | 0.37 (0.86) | 0.78 (0.33) | 0.90 (0.94) | 0.82 (0.44) | 0.95 (0.26) | 0.72 (0.45) | 0.41 (0.30) | **0.05 (0.03)** | 0.06 (0.12) |
|  | Women | 0.50 (0.11) | 0.20 (0.13) | 0.07 (0.18) | 0.87 (0.33) | 0.78 (0.84) | 0.40 (0.41) | 0.24 (0.51) | 0.53 (0.74) | 0.53 (0.69) |
| Fish and shellfish | Men | 0.25 (0.72) | 0.08 (0.29) | 0.16 (0.26) | 0.91 (0.86) | 0.76 (0.68) | 0.58 (0.59) | **0.005 (0.002)** | 0.16 (0.06) | 0.71 (0.91) |
|  | Women | 0.25 (0.25) | 0.64 (0.61) | 0.93 (0.94) | **0.02 (0.01**) | 0.24 (0.30) | 0.89 (0.56) | 0.93 (0.48) | 0.57 (0.07) | 0.65 (0.13) |

* *p*-Values in parenthesis: results excluding individuals reporting dietary changes in the past and misreporters of energy.
